# The 50s Cliff: A Decline in Perceptuo-Motor Learning, Not a Deficit in Visual Motion Perception

**DOI:** 10.1371/journal.pone.0121708

**Published:** 2015-04-13

**Authors:** Jie Ren, Shaochen Huang, Jiancheng Zhang, Qin Zhu, Andrew D. Wilson, Winona Snapp-Childs, Geoffrey P. Bingham

**Affiliations:** 1 Key Laboratory of Exercise and Health Sciences, Shanghai University of Sport, Shanghai, China; 2 Kinesiology and Health, University of Wyoming, Laramie, WY, United States of America; 3 Social, Psychological & Communication Sciences, Leeds Metropolitan University, Leeds, United Kingdom; 4 Psychological and Brain Sciences, Indiana University, Bloomington, IN, United States of America; University of Texas at Dallas, UNITED STATES

## Abstract

Previously, we measured perceptuo-motor learning rates across the lifespan and found a sudden drop in learning rates between ages 50 and 60, called the “50s cliff.” The task was a unimanual visual rhythmic coordination task in which participants used a joystick to oscillate one dot in a display in coordination with another dot oscillated by a computer. Participants learned to produce a coordination with a 90° relative phase relation between the dots. Learning rates for participants over 60 were half those of younger participants. Given existing evidence for visual motion perception deficits in people over 60 and the role of visual motion perception in the coordination task, it remained unclear whether the 50s cliff reflected onset of this deficit or a genuine decline in perceptuo-motor learning. The current work addressed this question. Two groups of 12 participants in each of four age ranges (20s, 50s, 60s, 70s) learned to perform a bimanual coordination of 90° relative phase. One group trained with only haptic information and the other group with both haptic and visual information about relative phase. Both groups were tested in both information conditions at baseline and post-test. If the 50s cliff was caused by an age dependent deficit in visual motion perception, then older participants in the visual group should have exhibited less learning than those in the haptic group, which should not exhibit the 50s cliff, and older participants in both groups should have performed less well when tested with visual information. Neither of these expectations was confirmed by the results, so we concluded that the 50s cliff reflects a genuine decline in perceptuo-motor learning with aging, not the onset of a deficit in visual motion perception.

## Introduction

Perceptuo-motor learning of coordinated actions is important at all ages. As children, we learn to perform the perceptuo-motor coordinations involved in daily tasks like dressing, eating, walking, or combing one’s hair. Then, older adults who have experienced stroke or injury are required to re-learn such coordinated actions or to learn new coordinations like walking with a walker or a cane or buttoning a shirt using the fingers of a single hand. Perceptuo-motor learning of coordinated rhythmic movements has been studied extensively using a model task introduced by Kelso [1], namely, coordinated bimanual rhythmic movement. This task entails rhythmic oscillatory movements of two limbs in coordination with one another (for instance, two hands oscillating about the wrist joints or two forearms moving about the elbow joints). Nearly everyone is able to perform two different modes of coordination readily and stably without special training or learning. The modes are described in terms of the relative phasing of the movements of the two limbs. One of the two easily produced modes is 0° relative phase. This means that each of the two limbs is doing the same thing as the other at the same time. So, for instance, the two wrists are flexed together and extended together. The other of the two modes is 180° relative phase and this means that the two limbs are moving oppositely at the same time. So, one wrist flexes while the other extends and vice versa. Modes other than these two typically cannot be produced spontaneously and stably, but they can be learned. So, for instance, without learning, one cannot typically perform bimanual rhythmic movement at 90° relative phase. In a 90° coordination, one wrist would be halfway through flexion (or extension) while the other wrist would be just beginning flexion (or extension) and vice versa. This coordination can be learned, however, with appropriate training [2].

In the original bimanual rhythmic coordination studies, a single person moved about two joints, each in a different limb, for instance, the left and right wrists. Subsequently, Schmidt et al. [3] found the same patterns of behaviour when two people each used vision to coordinate the rhythmic movement of a single limb with the rhythmic movement of a single limb of the other person. The two people could spontaneously produce stable 0° and 180° coordinated movement, but they could not produce other modes stably without training. See also [4]. This finding led to studies of visually controlled unimanual coordination in which a participant moved a single limb (for instance, a hand grasping and moving a joystick) to oscillate a dot in a computer display in coordination with another dot in the display that was oscillated by the computer [5]. This is called visual or unimanual coordination, because perceptual information about the coordination mode (that is, whether the dots are moving at 0° or 180°) is only available to vision. Such visual coordination also exhibits all the same patterns of behaviour as bimanual coordination. 0° and 180° coordination can be spontaneously and stably produced, but 90° coordination cannot be produced without special training and perceptuo-motor learning [6–7]. This and other research showed that coordination is perceptually mediated either by vision [5, 8–10], or for instance, in bimanual coordination with eyes closed, by haptic (or kinesthetic) perception [11].

Bimanual and unimanual coordination at 90° relative phase are ideal tasks for the study of perceptuo-motor learning because the tasks have been studied extensively so as to be well understood [12–14], yet they are truly novel to most participants. They are challenging, but doable, tasks for perceptuo-motor learning that can be performed by the young and old alike. Initial studies of older participants in their 70s attempting to learn these tasks, in comparison to younger participants in their 20s, showed that learning occurred at a slower rate [15–17]. This was consistent with findings in studies using other tasks to study perceptuo-motor learning [18–20]. However, in all these earlier studies, the actual learning rates were not measured. Coates et al. [21] studied age differences in learning rates. They investigated perceptuo-motor learning of a 90° relative phase in a visual unimanual coordination task and measured the learning rates of participants in their 20s compared with participants in their 70s and 80s. They found that the learning rates of the older participants were half those of the younger ones. The question raised by this finding was when and at what rate over the lifespan does the perceptuo-motor learning rate decline.

Coates et al. [22] used the same perceptuo-motor learning task as in their previous study [21] and measured learning rates of participants in their 20s, 30s, 40s, 50s, 60s, 70s, and 80s to discover the form of the change in perceptuo-motor learning ability over the lifespan. They found that a modest constant change occurred in perceptuo-motor learning rate from the 20s through the 40s, and then, during the 50s, a sudden larger drop in learning rate occurred, followed by little additional change in the 60s through the 80s. They called the sudden change the “50s cliff.”

Coates et al. [22] were not able to conclude that the 50s cliff necessarily reflected a genuine sudden decline in perceptuo-motor learning ability. The reason was that the visual coordination task entailed visual motion perception to control the coupling or relative phasing between the two moving dots in the display. Aging has been shown to negatively affect a variety of tasks requiring visual motion perception. See Anderson [23] for a review. For instance, the visual discrimination of speeds of motion is poor in older adults [24, 25]. Aging also yields poor performance in visually perceiving the 3D shape of objects viewed in Structure-From-Motion (SFM) displays [26, 27]. These results may reflect changes that have been found in aging of cortical function, such as reduced neuronal inhibition that leads to decreased center-surround antagonism in visual cortex for processing motion [28–30]. Thus, the 50s cliff might reflect these deficits in visual motion perception that emerge with aging rather than a sudden decline in perceptuo-motor learning as such.

The current study was designed to address whether the 50s cliff reflects a change in perceptuo-motor learning or a change in visual motion perception. We tested perceptuo-motor learning of 90° coordination by two different groups of participants in their 20s, 50s, 60s and 70s. Subsequently, each group was tested in one of two different bimanual coordination tasks. We used bimanual coordination tasks because they entailed the learning of a rhythmic coordination with a 90° relative phase without requiring use of visual information to guide the movements. Thus, both tasks included non-visual haptic (and therefore, kinesthetic) information about the coordinative movement being produced as participants grasped each of two joysticks and moved them. (In the previous studies by Coates et al., only a single joystick had been used because the task was visual unimanual coordination.) In one task (called ‘haptic’), no visual information about the movements was available. Only haptic information was available to the group of participants. In the other task (called ‘visual), both haptic and visual information about the movements was available to that group of participants. In this case, each of the two joysticks controlled the movement of a dot seen in a computer display so participants perceptually controlled the movements of the two dots both visually and haptically.

If a deficit in visual motion perception had been responsible for the 50s cliff found in the previous study, then we expected superior perceptuo-motor learning by older participants who performed the purely haptic bimanual coordination task. Accordingly, we would not expect the ‘50s cliff’ to appear in the results from this task. Also, we would expect the addition of visual information to result in poorer learning by older participants who performed the visual (and haptic) bimanual coordination task. Alternatively, if the 50s cliff reflected a genuine decline in perceptuo-motor learning, then we expected similar results for both groups and tasks, and the 50s cliff should be universal.

In addition, if the 50s cliff was produced by a deficit in visual motion perception, then we did not expect performance of older participants with the availability of visual information to be better than performance without it, that is, with only haptic information available. As an additional test of this expectation, we also tested participants in each group at baseline and post-test using the task practiced by the other group. Thus, participants in the haptic group also performed the visual task to test whether their performance might be worse than in the haptic task. The participants in the visual group also performed the haptic task to see whether their performance might be better than in the visual task. Alternatively, if there was no deficit in visual motion perception responsible for the observed performance levels, then participants in each group were simply expected to perform better in the task in which they had trained and at comparable levels.

Finally, if a deficit in visual motion perception was not a factor, then it was possible that performance might be better with the availability of visual information in addition to haptic information either because the visual information is better or simply because more information is better. In this case, especially if participants trained with both visual and haptic information, performance might be expected to be better with the availability and use of visual information.

## Method

### Ethics Statement

The study was approved by the Institutional Review Board at Shanghai University of Sport (SUS), CHINA, and written consent forms were obtained from all participants.

### Participants

A total of 96 adults were recruited from the residential communities surrounding the SUS campus through flyers disseminated during a wellness and health fair administered by the district government. Participants were recruited in four age groups: 24 were in their 20s (10 males and 14 females; mean age = 22); 24 were in their 50s (10 males and 14 females; mean age = 54); 24 were in their 60s (10 males and 14 females; mean age = 64); 24 were in their 70s (13 males and 11 females; mean age = 73). All participants were naïve to the experimental questions. Participants were screened and excluded from the study depending on their performance in an initial assessment session in which they performed coordinations of 0°, 180°, and 90°. The performance measure was the Proportion of Time on Task (PTT = % of the trial duration within an error bandwidth of the targeted phase, see [1, 7, 14] for details). Participants were invited to continue the study if their mean PTT performing 90° was less than 50%, and their mean PTT performing 180° was greater than that for 90° by at least 10%, otherwise, they were thanked for their interest and not included in the study. All participants were also screened for visual acuity using a standard Snellen chart (20/20 as normal or corrected to normal). They were also tested on the Upper Extremity Functional Scale [31] (the UEFS score≥80%) to make sure that they were able to move their two arms and hands and to perceive relevant visual and kinesthetic stimulation. Participants who did not pass these tests were not included in the study.

### Apparatus

**Fig 1:**
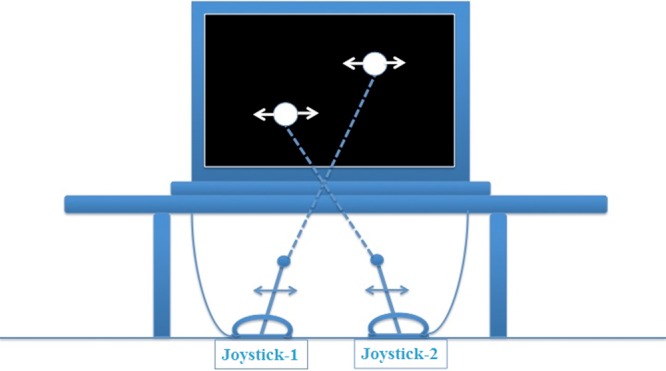
Illustration of apparatus used in the experiment. Illustration showing left and right joysticks that control the horizontal movements of the top and bottom dots on the computer display, respectively. The display sat on a shelf centered at eye height for the participant and the joysticks sat below this shelf where they could be grasped, but not seen, by the participant.

Participants sat on a stool (with adjustable height) facing a 15” PC laptop with its screen set to a resolution of 1024 x 768 and a refresh rate of 60Hz. Two Logitech Force 3D joysticks were connected via USB to the PC, one opposite the left shoulder of the participant and the other opposite the right shoulder. A custom-built shelf was set on the table on which the PC sat at eye height of the participant with the joysticks hidden underneath by a tablecloth covering the shelf. All participants reached and grasped the joysticks without seeing them. The computer displays showed two white dots on a black background, one dot above the other. The top dot was controlled by the left joystick, and the bottom dot by the right joystick (see Fig 1 for illustration). The amplitude of movement of each dot was 300 pixels, and each dot was 60 pixels in diameter at the viewing distance of 70 cm (yielding a movement that spanned ~7.5° visual angle). Stimulus presentation, data recording, and all data analyses were handled by a custom Matlab toolbox written by ADW, incorporating the Psychtoolbox [32].

### Procedure

Participants performed in two assessment sessions (baseline and post-test) and five training sessions, each occurring on a different day, following the timeline: baseline ➔ five training sessions ➔ post-test.

Participants in each age group were divided into two groups of 12 participants each for the learning of 90° during the training phase. One group, called ‘visual’, both visually and haptically perceived relative phase while learning 90°, and the other group, called ‘haptic’, only haptically perceived relative phase while learning 90°. The visual information was provided by moving dots in the display. Haptic information was of the hands moving the joysticks. Participants in the haptic group did not see dots moving in the display. Members of the two groups were tested in the same ways during assessment sessions with the exception of the initial demonstrations and practice trials. In each of the assessment sessions and for each of the different target relative phases (that is, 0°, 180° and 90°), participants first experienced an eight-second long demonstration of the target relative phase. The visual group viewed a display of two dots moving at the target relative phase. The haptic group experienced a coach passively moving the participant’s hands holding the joysticks to produce 90° while the participant’s eyes were closed. Then, participants in each group were given a practice trial (data not recorded) in which they grasped and moved joysticks to attempt to reproduce the pattern of the movement they had just experienced. During this attempt, they were provided with visual feedback indicating the accuracy of their movement performance. For the visual group, the moving dots turned from white to green when the relative phase being produced was within a bandwidth ± 20° of the target relative phase. For the haptic group, an unmoving dot on the screen turned from white to green when the relative phase being produced was within a bandwidth ± 20° of the target relative phase. After the demonstration and the practice trial, participants were tested in producing the target relative phase in each of five trials without visual feedback. Each trial lasted for 20 seconds. The target relative phases were: 0°, 180° and 90°, blocked and presented in that fixed order.

During the training phase, participants in each age and information group performed in five training sessions. In each of the five sessions, participants performed four blocks (three trials long) with a target relative phase of 90°. An eight-second long demonstration was provided at the beginning of each trial block. For the visual group, the demonstration was a display of two dots moving at a relative phase of 90°, and the following 3 training trials were provided with concurrent visual feedback, that is, when the participant moved at 90° within an error bandwidth, the person-controlled dots turned from white to green. For the haptic group, the demonstration involved manual guidance in which a coach passively moved the participant’s hands holding the joysticks to produce 90° while the participant’s eyes were closed. The concurrent feedback provided in the following three training trials was a single static dot in the center of screen that changed color from white to green when the participant actively moved the joysticks at 90° within an error bandwidth. The error bandwidth for the target relative phase was set to fade across sessions: ±30° in the first training session, ±25° the second, ±20° the third, ±15° the fourth, and ±10° the last.

The posttest assessment sessions were the same as the baseline assessment sessions with the exception that they were done twice for each group alternating the information made available in the second posttest session to be that of the other group in each case.

### Data Analysis

The two position time series from each trial were filtered using a low-pass Butterworth filter with a cut-off frequency of 10Hz and numerically differentiated to yield a velocity time series. These were used to compute a time series of relative phase, the key measure of coordination between the two joysticks.

To assess the stability of the coordination over the course of a trial, we used a measure of proportion of time on task (PTT). The measure is the proportion of time during a trial that the relative phase falls within a +/− 20° window of the target relative phase (e.g. 90°). We averaged PTT, for each participant, over the trials performed in a given condition. We chose PTT as the primary measure because, in human movement, stability is not independent of mean relative phase; so measures that simply assess overall movement variability (e.g. the standard deviation of mean relative phase or mean vector length) are confounded with the actual relative phase produced. Coordination stability at 90° can be artificially elevated if participants spend time at other locations (e.g. 0° or 180°), which they do as these locations are natural attractors [3], (see Wilson et al. [33] for an extended analysis of this problem). Proportion of time on task allows us to address this problem (see Snapp-Childs, et al. [14] for an explicit comparison of the two methods). It is simply the proportion of the relative phase time series that falls within the range of the target phase +/- a tolerance (e.g. of 20°), thus summarizing the data of interest (consistency and accuracy) and eliminating the confound. This measure ranges from 0–1 and validly measures stability of coordination at the required relative phase in a single number [7,8].

Means were computed for each trial and then across repeated trials performed by all participants within blocks. A learning score was computed by subtracting post-test means from paired baseline means.

First, we examined baseline performance at 0°, 180°, 90° visual and 90° haptic for all ages and both groups (visual and haptic) using a mixed design ANOVA with relative phase (0°, 180°, 90° visual and 90° haptic) as a repeated measure factor, and age (20-year olds, 50 year-olds, 60 year-olds, 70-year olds) and group (visual, haptic) as between-subjects factors. Next, we tested for learning using a mixed design ANOVA separately for each group with session (baseline, post-test) as a repeated measures factor and age (20-year olds, 50 year-olds, 60 year-olds, 70-year olds) as a between subjects factor. Then, we examined learning scores (the differences between PTT at baseline and post-test) for all ages and groups performing 90°. We used a factorial ANOVA with age (20-year olds, year-olds, 60 year-olds, 70-year olds) and group (visual, haptic) as between-subjects factors. To examine interactions, t-tests (2-tailed) were used. We also tested transfer from the task and information used during training (visual or haptic) to the other task and information (haptic or visual, respectively). To do this, we added task (same, different) as a repeated measure to the previous ANOVA design and included the different task scores which were 90° haptic for the visual group and 90° visual for the haptic group.

We also examined learning rates. Exponential functions were fitted to learning curve data. The functions were of the form:

$$\text{PPT=a}e^{\frac{\text{-b}}{\text{S}}}$$ (1)

where PTT is ‘Proportion of Time on Task’, S is session (1 = baseline, 2–6 = training, and 7 = post-test), and a and b are parameters. The function was fitted to the data for each participant. First, the PTT means and session numbers were transformed as follows:

$$\text{PTT}\to \text{ln}\left(\text{PTT}\right)\text{ and S}\to \frac{\text{1}}{\text{S}}$$

Then, least squares linear regression was used to fit a line to the relation between the two sets of transformed values, separately for each participant. The slopes and intercepts yielded by these fits were used to derive the parameters a and b in Equation (1). Then, we computed the first derivative of the function in Equation (1) as follows:

$$\frac{\left(\text{ab}\right)}{{\text{S}}^{\text{2}}}{\text{e}}^{\frac{\text{-b}}{\text{S}}}$$ (2)

We then evaluated Equation (2) at Session 1 (S = 1) to get a value for learning rate for each participant. Negative values were set to 0 (16% overall). Finally, we performed a factorial ANOVA on these learning rates with age (20s, 50s, 60s, 70s) and group (visual, haptic) as factors. Also, all combinations of ages were compared taken two at a time.

## Results

Proportion of Time on Task (PTT) was measured for each trial. This was the portion of the time in each trial during which the produced relative phase was within +/–20° of the 90° target phase. For each participant and block of trials within condition, we computed the mean PTT over trials within a block. All subsequent analyses were performed on these mean PTT scores.

### Baseline

At baseline, the level of performance in each of the two groups (visual and haptic) should not have been different. On the other hand, the level of performance in both groups should have been significantly worse when participants attempted to produce 90° relative phase as compared to 0° or 180° relative phase, because people are able to do 0° and 180° intrinsically, but have to learn to be able to do 90° and because we meant to exclude from participation people who had already learned to do 90°. In addition, baseline performance might have varied as a function of age level or task (that is, 90° visual versus 90° haptic).

To address these possibilities, we performed a mixed design Analysis of Variance (ANOVA) on mean Proportion of Time on Task (PTT) at baseline with group (visual, haptic) and age (20, 50, 60, 70) as between-subjects factors and phase (0°, 180°, 90° visual, 90° haptic) as a repeated-measures factor. The result yielded main effects of age (F(3,88) = 9.4, p < 0.001, η^2^ = 0.02) and phase (F(3,264) = 864.4, p < 0.001, η^2^ = 0.83), but not of group (p > 0.1). The mean PTT was greater for younger participants: 20s = 0.47; 50s = 0.42; 60s = 0.39; and 70s = 0.39. The mean PTT was greater for 0° (0.62) and 180° (0.66) as compared with 90° visual (0.20) or 90° haptic (0.19). In addition, there was a significant age by phase interaction (F(9,264) = 3.4, p < 0.001, η^2^ = 0.01). While mean PTT was constant across ages at 0°, it varied across ages for 180° and 90°. Furthermore, there appeared to be a difference between 90° visual and 90° haptic, especially for participants in their 20s.

To test this latter possibility, we performed the ANOVA with only two levels of phase: 90° visual and 90° haptic. The result again yielded main effects for age (F(3,88) = 11.4, p < 0.001, η^2^ = 0.24) and phase (F(1,88) = 4.2, p < 0.05, η^2^ = 0.005) as well as an age by phase interaction (F(3,88) = 7.3, p < 0.001, η^2^ = 0.03). Overall, 90° visual was greater than 90° haptic as shown in Table 1. As also shown, 90° visual only yielded better performance for participants in their 20s (who were also better overall). We performed paired t-tests (2-tailed) comparing 90° visual and 90° haptic at each age. Only the 20s yielded a significant difference (t(23) = 3.6, p < 0.002). Other ages did not reach significance (p > 0.2 or more).

**Table 1:** Mean Proportion of Time on Task at baseline for each age and information group.

	20s	50s	60s	70s	Overall
**90° visual:**	0.29	0.19	0.17	0.15	0.20
**90° haptic:**	0.23	0.18	0.15	0.15	0.18

Finally, we tested for potential novelty effects in the two learning conditions, 90° visual and 90° haptic. In each condition, we performed a multiple regression testing for an increasing trend in the PTT measure over trials in baseline, with age and age by trial interaction included as factors. In the 90° visual condition, the analysis was significant (F(3, 476) = 74.1, p < 0.001, r^2^ = 0.32), but only age was significant (p < 0.001, t = 6.2). Both trial (p > 0.4) and the interaction (p > 0.8) failed to reach significance. In the 90° haptic condition, the analysis was significant (F(3, 476) = 20.6, p < 0.001, r^2^ = 0.12), but again only the age was significant (p < 0.001, t = 3.2). Both trial (p > 0.7) and the interaction (p > 0.9) failed to reach significance.

Overall the analyses of baseline data showed no differences between the visual and haptic groups, but clear differences as a function of both age and the presence or absence of visual information. In respect to age, participants in their 20s and 50s did better than those in their 60s and 70s. Furthermore, participants in their 20s were able to perform better with visual information than without it, but participants at other ages did not exhibit this advantage. There was no evidence of novelty effects.

### Baseline versus Post-test at 90° Relative Phase

We tested whether participants in each of the two groups (visual and haptic) improved in their performance of 90° relative phase as a result of training and whether any such improvement varied as a function of age, that is, whether older participants exhibited less of an increase in the Proportion of Time on Task (PTT) than did younger participants. We compared mean PTT scores at post-test to baseline scores. Only the data from the task experienced in training were analysed here for each group, respectively. For the visual group, the task was 90° visual and for the haptic group, it was 90° haptic.

We performed a mixed design ANOVA on mean PTT data with group (visual, haptic) and age (20, 50, 60, 70) as between-subjects factors and session (baseline, post-test) as a repeated-measures factor. The results yielded main effects of age (F(3,88) = 35.6, p < 0.001, η^2^ = 0.30) and session (F(1,88) = 99.6, p < 0.001 η^2^ = 0.18). There were also significant interactions of age by session (F(3,88) = 12.6, p < 0.001, η^2^ = 0.07) and of age by group (F(3,88) = 4.9, p < 0.005, η^2^ = 0.04). The PTT means relevant to this latter interaction are shown as the overall scores by age in Table 2 (for the visual group) and Table 3 (for the haptic group). We performed unpaired t-tests (2 tailed) to test the difference between groups at each age. Only the difference for the 20s reached significance (t(22) = 2.5, p < 0.02. All other ages were p > 0.3. This was the result to be expected given the findings in the analysis of baseline data, namely that the availability of visual information was advantageous, but only for participants in their 20s. However, this advantage was for performance overall, not for learning. Younger participants performed better than older participants. The PTT means by age were as follows: 0.34 for 20s, 0.24 for 50s, 0.20 for 60s, and 0.17 for 70s. Nevertheless, older participants improved with training, We performed paired t-tests (2-tailed) comparing Baseline and Post-test for each age with results as follows: for the 20s: t(23) = 7.4, p < 0.001; for the 50s: t(23) = 5.6, p < 0.001; for the 60s: t(23) = 2.2, p < 0.04; for the 70s: t(23) = 4.1, p < 0.001. One participant among those in 60-year old age group performed substantially less well in the Post-test. These tests were all significant, p < 0.05, with a Holm-Bonferroni correction [34].

**Table 2:** Visual group: Mean Proportion of Time on Task for each age group at baseline and post-test together with overall means.

	20	50	60	70	Overall
**Baseline:**	0.28	0.19	0.15	0.13	0.19
**Post-test:**	0.50	0.32	0.20	0.19	0.30
**Overall:**	0.39	0.26	0.18	0.16	0.24

**Table 3:** Haptic group: Mean Proportion of Time on Task for each age group at baseline and post-test together with overall means.

	20	50	60	70	Overall
**Baseline:**	0.21	0.18	0.20	0.15	0.19
**Post-test:**	0.40	0.27	0.24	0.22	0.28
**Overall:**	0.30	0.22	0.22	0.18	0.24

### Testing learning scores

Because participants exhibited different performance levels in baseline as a function of age level and task (90° visual versus 90° haptic), we computed learning scores to provide a measure of the change in performance level as a result of training, independent of the initial baseline levels of performance. Learning scores were computed by subtracting Baseline from Post-test PTT scores. We analysed learning scores for the tasks in which participants in each group had trained, that is, 90° visual for the visual group and 90° haptic for the haptic group. The scores were tested in a multi-factor ANOVA with age (20, 50, 60, 70) and group (visual, haptic) as between-subjects factors. The results yielded only a main effect of age (F(3, 88) = 12.6, p< 0.001, η^2^ = 0.29). There was no difference between the visual and haptic groups.

We used the confidence interval approach to the two one-sided test procedure to infer equivalence of the visual and haptic groups in respect to learning. In this procedure, equivalence is established if the designated confidence interval (for α = 0.05, the CI = (1–2 α)×100 = 90%) for the mean difference between groups is contained within the equivalence margin or (-δ, δ) interval [35]. For this experiment, the mean difference between groups was obtained by subtracting the haptic group’s mean performance from the visual group’s mean performance (so negative numbers reflect the haptic group being superior to the visual group). The (-δ, δ) interval was set at (-0.10, 0.10). We chose this (-δ, δ) because this approximately reflects differences in the total proportion measure found to be reliably different in this and many previous studies. The mean difference between groups and the confidence intervals were: 0.022 ± 0.032 (-0.010, 0.054). Because these range limits were half the δ–levels required to accept the null hypothesis (that is, lack of equivalence), we concluded that trained visual and haptic performances were equivalent.

**Fig 2:**
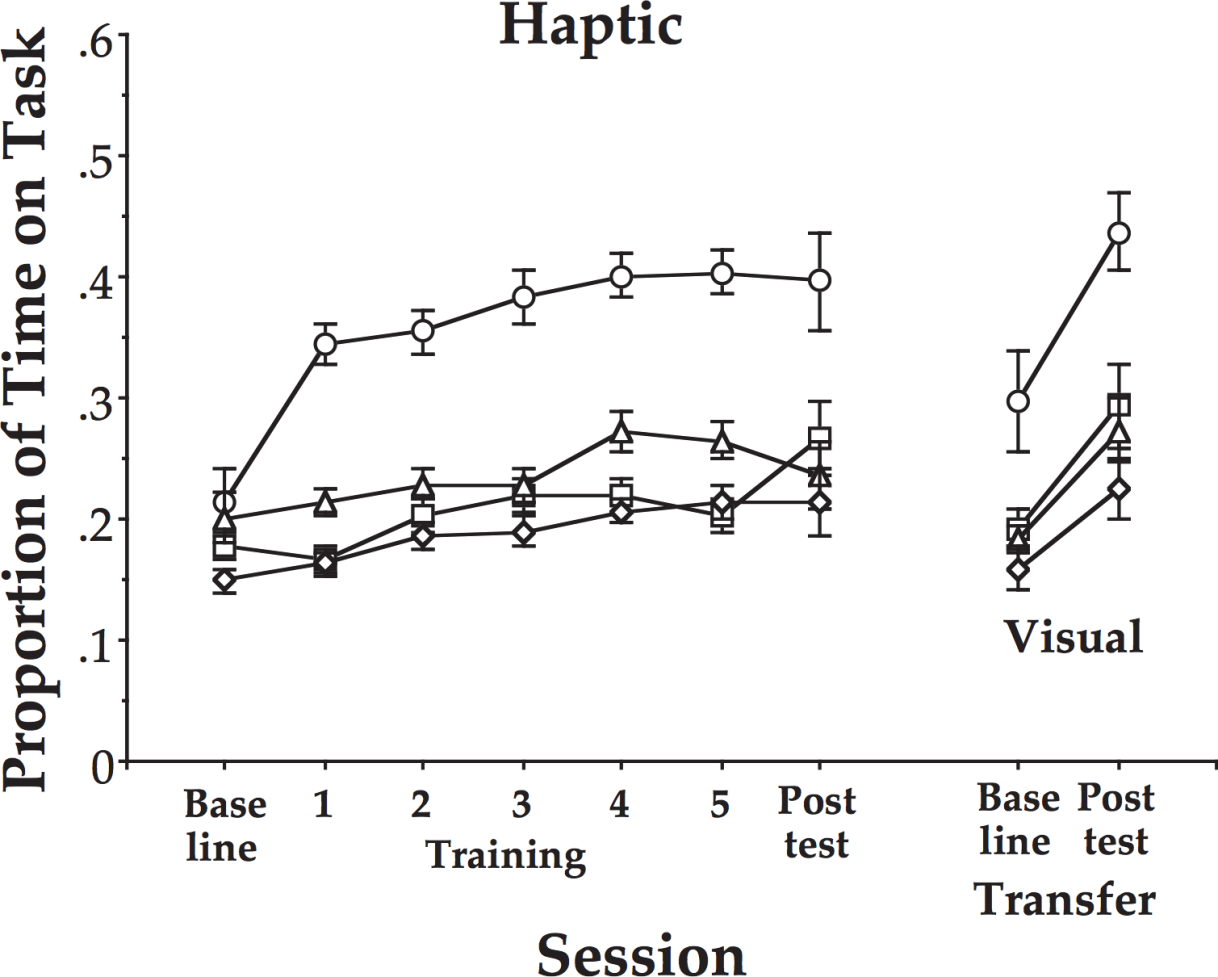
Proportion of time on task for the haptic group across age, and assessment sessions. Mean proportion of time spent within 20° of the 90° target mean relative phase across the baseline, 5 training sessions, and post-test session for the haptic group performing the task with haptic information only, followed by the transfer task with visual information at baseline and post-test for all 4 age groups. 20s: circles; 50s: squares; 60s: triangles; 70s: diamonds. Error bars are standard errors.

As shown in Fig 2, younger participants exhibited larger learning scores than older participants. Independent (2-tailed) t-tests yielded significant differences (t(46) > 2.2, p < 0.05 or better with Holm-Bonferroni correction [35]) between 20s and 50s, 60s and 70s, between 50s and 60s and 70s, but not between 60s and 70s (p > 0.4).

The 50s cliff appears in Fig 2 just as it did in Coates et al. [22]. If the 50s cliff reflected a deficit in visual motion perception, then the expectation would be that the older participants in the visual group should have improved less than those in the haptic group, which in turn, should not have exhibited the 50s cliff. The results failed to confirm this expectation. The two groups exhibited equal amounts of learning and both exhibited the 50s cliff. This supported the conclusion that the 50s cliff reflects a genuine decline in perceptuo-motor learning.

### Testing transfer of learning to tasks using other information

Next, we tested whether participants, that are trained in one task (using either visual or haptic information), and then are tested in the other task (using haptic or visual information, respectively), exhibit the same learning scores in the transfer task. If the 50s cliff reflects an age dependent deficit in visual motion perception, then the expectation would be that performance would consistently be worse for older participants when using visual information.

We performed a mixed design ANOVA on learning scores with age (20, 50, 60, 70) and group (visual, haptic) as between-subjects factors and task (same, different) as a repeated-measures factor. For the visual group, same was 90° visual and different was 90° haptic. For the haptic group, same was 90° haptic and different was 90° visual. The results yielded main effects of age (F(3,88) = 8.9, p < 0.001, η^2^ = 0.19) and task (F(1, 88) = 4.4, p < 0.05, η^2^ = 0.007). There was also a significant interaction of age by task (F(3,88) = 2.9, p < 0.05, η^2^ = 0.02). As shown in Table 4, performance was better when the task was the same as in training, but only for younger participants. A paired t-test (2-tailed) was performed to test same versus different for each age. Only 20s was significant (t(23) = 2.3, p < 0.05).

**Table 4:** Learning scores for each age group and task.

	20	50	60	70	Overall
Same:	0.20	0.11	0.06	0.06	0.11
Different:	0.14	0.10	0.06	0.05	0.09

The availability and use of visual information did not yield a decrement in learning for older participants. Older participants exhibited equal amounts (with the means equal or within a single percentage point) of improvement in performance with learning when tested using visual or only haptic information (although the improvement was less than their younger counterparts). This supports the conclusion that the 50s cliff reflects a genuine decline in perceptuo-motor learning and not a deficit in visual motion perception. Furthermore, younger participants exhibited superior performance when tested with the same information with which they had trained. This occurred both when training with visual and with haptic information. Thus, there was not an improvement that was specific to the type of information. The improvement was specific to the training.

### Learning rates

Next, we tested whether learning rates exhibited a decrease as age increased with a steep decline between 50 and 60 years of age, that is, a 50s cliff comparable to that in previous findings [22].

**Fig 3:**
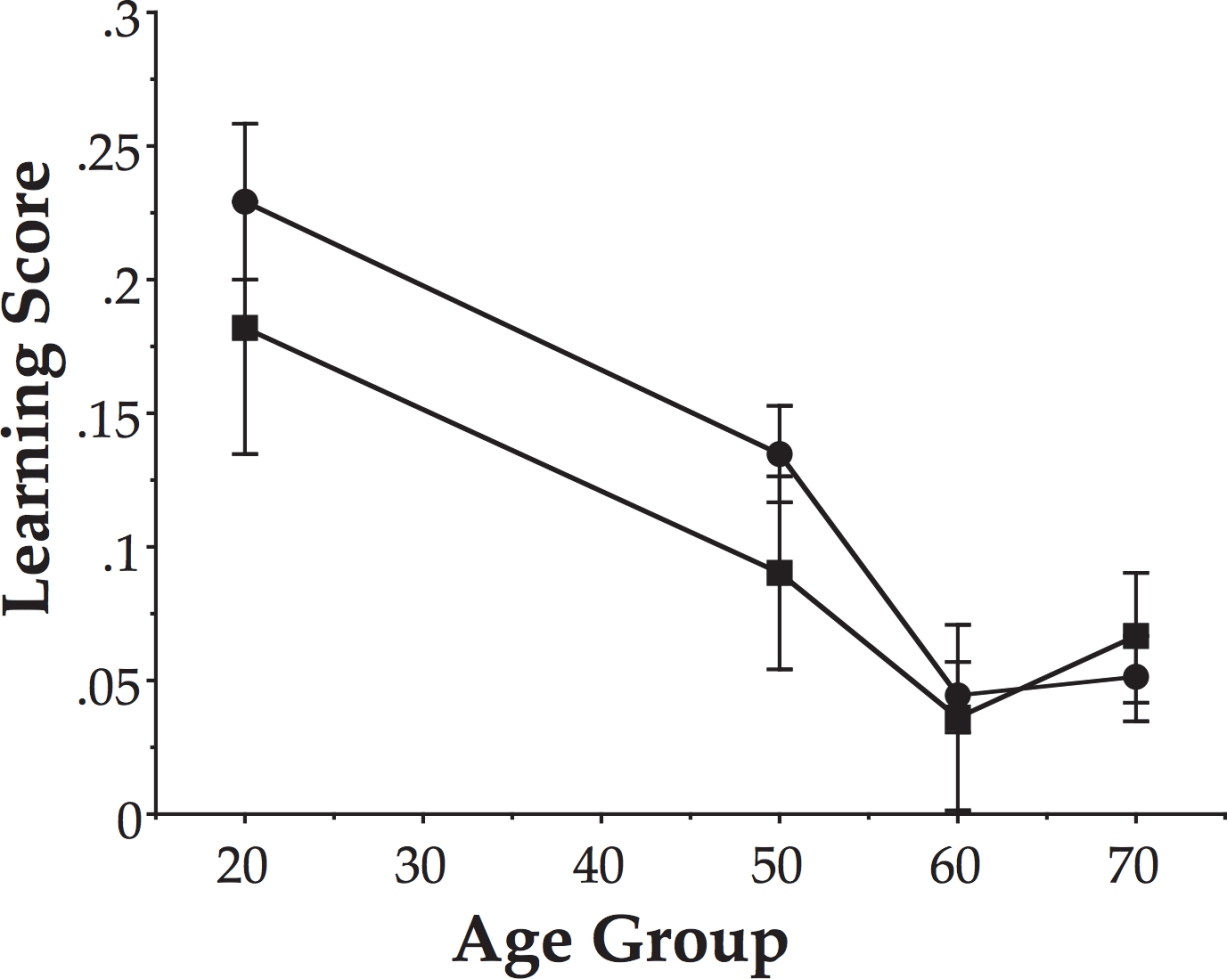
Mean learning scores. Mean learning scores for all 4 age groups and both training groups. Scores computed as difference of post-test and baseline proportion of time on task. Error bars are standard errors. Visual group: circles; Haptic group: squares.

**Fig 4:**
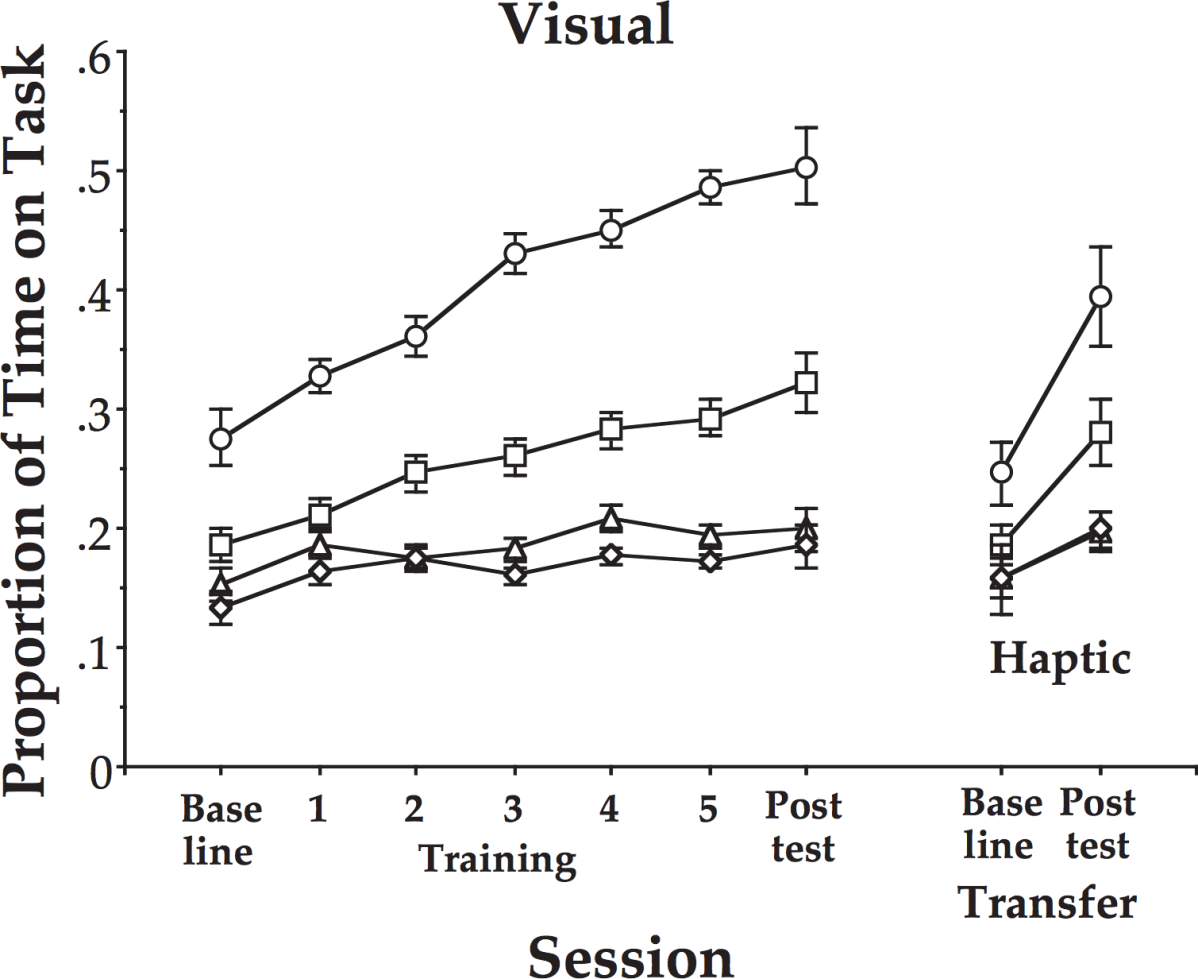
Proportion of time on task for the visual group across age, and assessment sessions. Mean proportion of time spent within 20° of the 90° target mean relative phase across the baseline, 5 training sessions, and post-test session for the visual group performing the task with visual information, followed by the transfer task with haptic information only at baseline and post-test for all 4 age groups. 20s: circles; 50s: squares; 60s: triangles; 70s: diamonds. Error bars are standard errors.

Fig 3 shows the mean learning curve for each of the age groups performing 90° relative phase across all sessions with visual information. Fig 4 shows the mean learning curve for each of the age groups performing 90° relative phase across all sessions with only haptic information. As described in the methods, exponential functions were fitted to these curves, including baseline, training and post-test sessions. The results were used to evaluate the derivative of the functions at Session 1 to derive an estimate of the learning rate for each participant. (Two of the oldest participants, 57 and 58 years respectively, of the 50s haptic group yielded significant positive slopes reflecting the fact that their mean PTT for training sessions were all less than for baseline. These two participants were excluded from the following analysis.) The learning rates were then tested using a factorial ANOVA with age (20s, 50s, 60s, 70s) and group (visual, haptic) as between-subjects factors. The results yielded a main effect of age (F(3, 86) = 15.1, p < 0.001, η^2^ = .33). Neither group nor the interaction was significant (p > 0.4). We performed independent t-tests (2-tailed) with Holm-Bonferroni corrections comparing age groups two at a time and found 20s different from 50s, 60s, and 70s (t(46) > 2.5, p < 0.02 or better), 50s different from 60s and 70s (t(44) > 2.5, p < 0.05 or better), but 60s was not different from 70s (p > 0.4).

**Fig 5:**
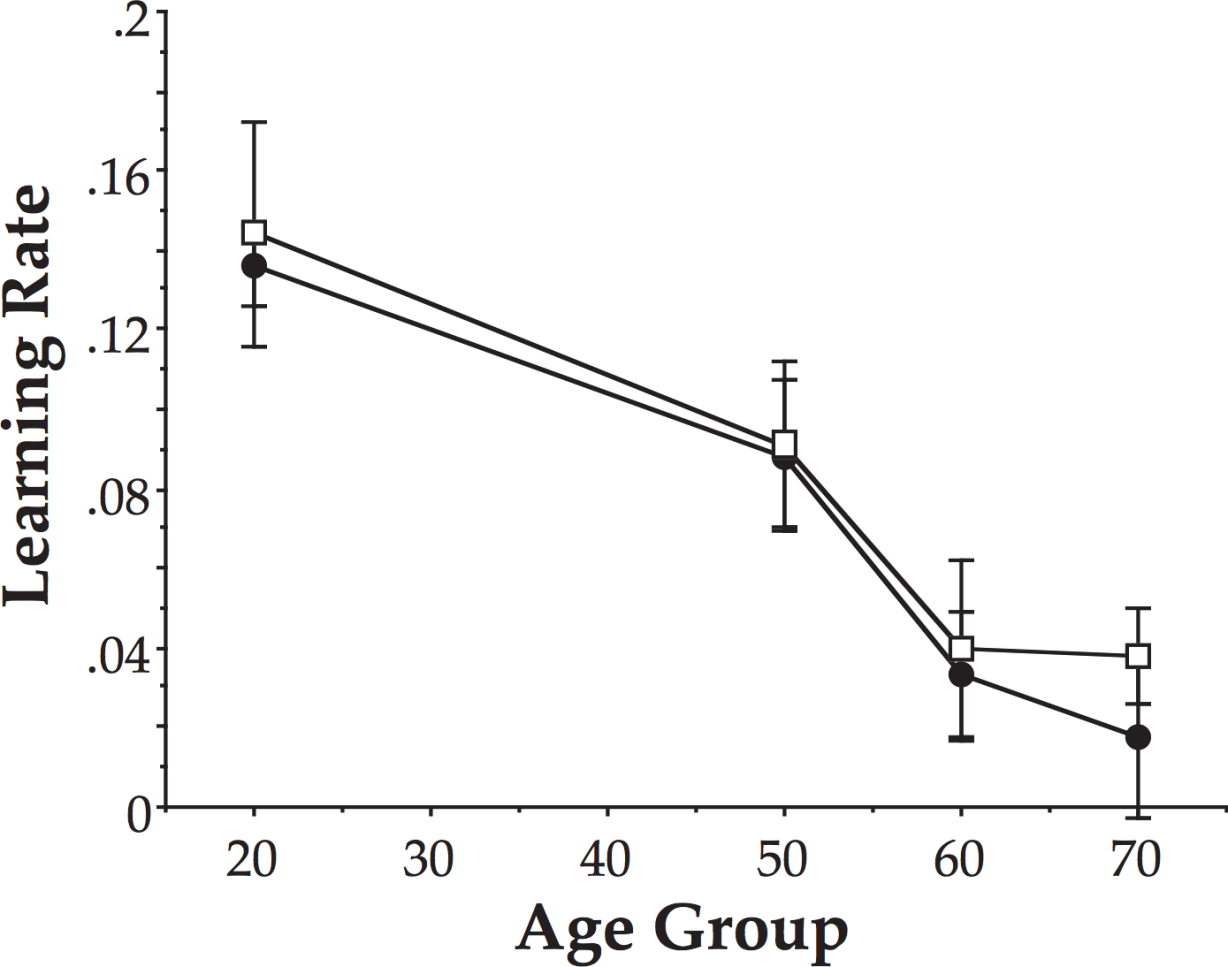
Mean learning rates. Mean learning rates for all 4 age groups and both training groups. Error bars are standard errors. Visual group: filled circles; Haptic group: open squares.

As shown in Fig 5, these learning rates exhibited nearly (and sometimes exactly) the same mean values, and thus, the same pattern for both groups (visual and haptic), including the 50s cliff, which appears as it did in the previous study by Coates et al. [22].

## Discussion

Previously, Coates et al. [21] had investigated changes in perceptuo-motor learning rates with aging. They tested participants in their 20s compared with participants over 60 (60s, 70s, and 80s) using the visual unimanual rhythmic coordination task. Coates et al. found that the learning rates of the older participants were half those of the younger participants in their 20s.

Subsequently, Coates et al. [22] investigated the way these learning rates change over the lifespan. Using the same task, they tested participants in all the decades from the 20s through to the 80s. They found a steady but modest gradual decline in learning rate (by about a fifth) as age progressed from the 20s up through the 40s. Then, from the 50s to 60s, the rate dropped suddenly and significantly to half of the original rate. Then, the learning rates remained steady through to the 80s. The sudden drop was called the ‘50s cliff’. This was a startling result that was potentially of great significance. Perceptuo-motor learning can be of great importance for older people who need to re-learn old movement skills or learn news ones to recover from and adapt to injury or stroke, for instance. However, Coates et al. [22] were unsure whether the 50s cliff reflected a genuine decline in perceptuo-motor learning rates as a function of age or whether instead, it reflected a deficit in visual motion perception that emerges with aging. They noted that their coordination task entailed visual motion perception as essential to performance, because only visual information about the relative phase relation between the moving dots was available to control the dots to try to produce the target phase of 90°. Investigations have shown that when visual motion perception is performed by observers over 60 years of age, the performance is poor for certain tasks including 3D shape perception from motion (with any amount of noise in the motion display) [24, 25] and speed discrimination [26, 27]. Thus, it was possible that the 50s cliff reflected a decline in motion perception ability rather than in perceptuo-motor learning ability.

The research reported in the current paper was designed to discover whether the 50s cliff reflected change in motion perception or perceptuo-motor learning. We tested participants in age groups that would reveal the 50s cliff including participants in their 20s, 50s, 60s and 70s. Instead of the visual unimanual coordination task, we employed a bimanual coordination task that could be performed either with or without the availability of visual information about the coordinated movements. Participants used their left and right hands to grasp left and right joysticks and then move them to produce oscillatory movements with specific relative phase relationships between the moving joysticks. Different groups of participants in each age range performed the task either with haptic information only or with both haptic and visual information about the movements and the phase relationship being produced. Haptic information about relative phase was available from the hands moving the joysticks. These could not be seen, however, because they were behind a shelf that occluded them. Half the participants performed with visual information made available because the joysticks each controlled the movement of a dot in a computer display, each of the two dots moving back and forth, one dot above the other in the display. One group of participants trained at performing 90° relative phase using only haptic information about relative phase. Both before and after training they were tested both with and without visual information in addition to the haptic information about phase. The other group trained using both visual and haptic information about relative phase. Both before and after training, they were tested both with and without the visual information in addition to the haptic information.

If the 50s cliff was caused by a deficit in visual motion perception with aging, then we expected two results in the performance of older participants. First, we expected the amount of learning with training to be less when training was done with visual information than without it. In this case, we would not expect the 50s cliff to appear in the results for the group that trained only with haptic information. Second, we expected performance with visual information to be worse in general, even if the (older) participants trained using only haptic information. On the other hand, if we found the amount of learning to be comparable both with and without visual information and the 50s cliff appeared in the results for both groups, then this would suggest that the 50s cliff reflects a genuine decrement in perceptuo-motor learning. Likewise, if an older participant’s performance after training was either equally good using either type of information, or better simply using the information with which the participant had trained (that is, better with haptic, if trained with haptic, and better with visual if trained with visual), then this also would support the conclusion that the 50s cliff was not due to a visual motion perception deficit and instead, reflects a decrement in perceptuo-motor learning with aging. Finally, if performance of older participants was generally better, both before and after training, with the additional information supplied by vision, then again the 50s cliff could not have been due to a deficit in visual motion perception that emerges with aging and must reflect a genuine decrement in learning.

The results supported the conclusion that the 50s cliff reflects a genuine decline in perceptuo-motor learning that occurs with aging. First, we found no difference in the amount of learning as a function of the information used during training. Analysis of learning scores yielded no effects of training groups that trained with vision or only haptic information. There were differences in performance at baseline. Younger participants did better overall at baseline and in addition, younger participants did better at baseline with visual information available. However, the amount of learning achieved as a result of training with either haptic plus visual or only haptic information about relative phase was not different at any age. Learning occurred at all ages, although younger participants exhibited greater amounts of learning overall (independent of the information used during training), especially participants in their 20s. So again, older participants learned equally well when they trained using visual versus haptic information. Finally, the 50s cliff appeared in the results for both training groups and in particular, in the group that trained using only haptic information.

Second, we found that younger participants did better when tested, after training, with the same information that they had used during training. There was no difference for older participants as a function of the type of information with which they were tested (independent of what information they had used during training). Thus, older participants did not perform worse with visual information. They also did not perform better with the addition of visual information. For all participants both young and old, the amount of learning was independent of the type or amount of information available during training. Younger participants (but not older ones) performed better after training when tested with the type and amount of information with which they had trained. Thus, the learning appeared to be more information specific for younger, but not older, participants. On the other hand, younger participants exhibited substantially greater learning as had been found in previous studies [21, 22].

Finally, when we measured and analysed learning rates, we found that the pattern of results was the same as that found previously by Coates et al. [22] with no differences between the two groups in the current study, that is, visual versus haptic. Learning rates decreased from 20s to 50s and were lowest for 60s and 70s. Both groups exhibited the 50s cliff pattern, that is, after more gradual decline in learning from 20s to 50s, a large decrease occurred between 50s and 60s, after which, learning rates remained constant. Again, there was no difference between the two groups showing that the 50s cliff cannot be merely the result of poor visual motion perception.

Because we only tested younger participants in their 20s and 50s, and not also 30s and 40s as in the previous study [22], it remains possible that the steeper decline in learning rate might have started earlier in case of bimanual coordination with or without vision. We cannot rule out the possibility that learning rates remained constant until, for instance, the 40s at which point the rate of decline evident between 50 and 60 might have begun. Nevertheless, the cliff would remain and extend through the 50s. The current and previous [22] results show that the decade of the 50s is a period of steep decline in perceptuo-motor learning rates.

Collectively, the evidence supports the conclusion that the 50s cliff is, in fact, a decline in perceptuo-motor learning and not merely a decline in motion perception abilities.

## Supporting Information

S1 Dataset (ZIP)

S2 Dataset (ZIP)

S3 Dataset (ZIP)

S4 Dataset (ZIP)

S5 Dataset (ZIP)

S6 Dataset (ZIP)

S7 Dataset (ZIP)

S8 Dataset (ZIP)

S9 Dataset (ZIP)

S10 Dataset (XLSX)

## References

[1] Kelso JAS (1981) On the oscillatory basis of movement. Bull Psychon Soc 18:63

[2] Zanone PG, Kelso JAS (1992) Evolution of behavioral attractors with learning: Nonequilibrium phase transitions. J Exp Psychol Hum Percept Perform 18(2):403–421 1593227 10.1037//0096-1523.18.2.403

[3] Schmidt RC, Carello C, Turvey MT (1990) Phase transitions and critical fluctuations in the visual coordination of rhythmic movements between people. J Exp Psychol Hum Percept Perform 16:227–247 2142196 10.1037//0096-1523.16.2.227

[4] Temprado JJ, Swinnen SP, Carson RG, Tourment A, Laurent M (2003) Interaction of directional, neuromuscular and egocentric constraints on the stability of preferred bimanual coordination patterns. Hum Move Sci 22:339–363 10.1016/s0167-9457(03)00049-6 12967762

[5] Wimmers RH, Beek PJ, Wieringen PCW (1992) Phase transitions in rhythmic tracking movements: A case of unilateral coupling. Hum Move Sci 11(1–2):217–226

[6] Wilson AD, Snapp-Childs W, Coats RO, Bingham GP (2010) Learning a coordinated rhythmic movement with task-appropriate coordination feedback. Exp Brain Res 205(4):513–520 10.1007/s00221-010-2388-y 20703872

[7] Wilson AD, Snapp-Childs W, Bingham GP (2010) Perceptual Learning Immediately Yields New Stable Motor Coordination. J Exp Psychol Hum Percept Perform 36(6):1508–1514 10.1037/a0020412 20731515

[8] Bingham GP, Schmidt RC, Zaal F (1999) Visual perception of the relative phasing of human limb movements. Percept Psychophys 61(2): 246–258 10089759 10.3758/bf03206886

[9] Mechsner F, Kerzel D, Knoblich G, Prinz W (2001) Perceptual basis of bimanual coordination. Nature 414:69–73 11689944 10.1038/35102060

[10] Zaal F, Bingham GP, Schmidt RC (2000) Visual perception of relative phase and phase variability. J Exp Psychol Hum Percept Perform 26(3):1209–1220 10884018 10.1037//0096-1523.26.3.1209

[11] Wilson AD, Bingham GP, Craig JC (2003) Proprioceptive perception of phase variability. J Exp Psychol Hum Percept Perform 29(6):1179–1190 14640837 10.1037/0096-1523.29.6.1179

[12] Bingham GP (2004) Another timing variable composed of state variables: Phase perception and phase driven oscillators. In: Hecht H, Savelsbergh G (eds) Theories of Time-to-Contact. North Holland, Amsterdam

[13] Snapp-Childs W, Wilson AD, Bingham GP (2011) The stability of rhythmic movement coordination depends on relative speed: the Bingham model supported. Exp Brain Res 215:89–100 10.1007/s00221-011-2874-x 21952789

[14] Kelso JAS (1995) Dynamic Patterns: The Self-Organization of Brain and Behavior. MIT Press, Cambridge, MA

[15] Serrien DJ, Swinnen SP, Stelmach GE (2000) Age-related deterioration of coordinated interlimb behaviour. J Gerontol 55B(5): 295–303 10.1093/geronb/55.5.p295 10985294

[16] Swinnen SP, Verschueren SMP, Bogaerts H, Dounskaia N (1998) Age-related deficits in motor learning and differences in feedback processing during the production of a bimanual coordination pattern. Cognitive Neuropsych 15(5):439–466 10.1080/026432998381104 28657466

[17] Wishart LR, Lee TD, Cunningham SJ, Murdoch JE (2002) Age-related differences and the role of augmented visual feedback in learning a bimanual coordination pattern. Acta Psychol 110:247–263 12102108 10.1016/s0001-6918(02)00036-7

[18] Voelcker-Rehage C (2008) Motor-skill learning in older adults—a review of studies on age-related differences. Eur Rev Aging Phys Activ 5:5–16

[19] Ghisletta P, Kennedy KM, Rodrigue KM, LIndenberger U, Raz N (2010) Adult age differences and the role of cognitive resources in perceptual-motor skill acquisition: application of a multilevel negative exponential model. J Gerontol B Psychol Sci Soc Sci 65B(2): 163–173 10.1093/geronb/gbp126 20047985 PMC2981445

[20] Panzer S, Gruetzmacher N, Fries U, Krueger M, Shea CH (2011) Age-related effects in interlimb practice on coding complex movement sequences. Hum Mov Sci 30(2): 459–474 21349597 10.1016/j.humov.2010.11.003

[21] Coats RO, Snapp-Childs W, Wilson AD, Bingham GP (2013) Perceptuo-motor learning rate declines by half from 20s to 70/80s. Exp Brain Res 225:75–84 10.1007/s00221-012-3349-4 23212470

[22] Coats RO, Wilson AD, Snapp-Childs W, Fath AJ, Bingham GP (2014) The 50’s cliff: Perceptuo-motor learning across the lifespan. PLoS ONE 9(1): e85758 10.1371/journal.pone.0085758 24475051 PMC3901653

[23] Anderson GJ (2012) Aging and vision: Changes in function and performance from optics to perception. WIREs Cogn Sci 3(3):403–410 10.1002/wcs.1167 PMC3424001 22919436

[24] Norman JF, Ross HE, Hawkes LM, Long JR (2003). Aging and the perception of speed. Perception 32:85–96 12613788 10.1068/p3478

[25] Snowden RJ, Kavanagh E (2006) Motion perception in the ageing visual system: Minimum motion, motion coherence, and speed discrimination thresholds. Perception 35:9–24 16491704 10.1068/p5399

[26] Norman JF, Dawson TE, Butler AK (2000) The effects of age upon the perception of depth and 3-D shape from differential motion and binocular disparity. Perception 29: 1335–1359 11219988 10.1068/p3111

[27] Norman JF, Clayton AM, Shular CF, Thompson SR (2004) Aging and the Perception of Depth and 3-D Shape from Motion Parallax. Psychol Aging 19: 506–514 15383000 10.1037/0882-7974.19.3.506

[28] Betts LR, Taylor CP, Sekuler AB, Bennett PJ (2005) Aging reduces center-surround antagonism in visual motion processing. Neuron 45: 361–366 15694323 10.1016/j.neuron.2004.12.041

[29] Liang Z, Yang Y, Li G, Zhang J, Wang Y (2010). Aging affects the direction selectivity of MT cells in rhesus monkeys. Neurobiol of Aging 31(5): 863–873 10.1016/j.neurobiolaging.2008.06.013 18674846

[30] Nedelko V, Hassaa T, Hamzeib F, Weiller C, Binkofski F (2010) Age-independent activation in areas of the mirror neuron system during action observation and action imagery. A fMRI study. Restor Neurol Neuros 28:737–747 10.3233/RNN-2010-0542 21209489

[31] Stratford PW, Binkley JM, Stratford DM (2001) Development and initial validation of the upper extremity functional index. Physiotherapy Canada 53(4): 259–267

[32] Wilson AD, Tresilian J, Schlaghecken F (2011) The masked priming toolbox: an open-source MATLAB toolbox for masked priming researchers. Beh Res Meth 43: 210–214 10.3758/s13428-010-0034-z 21287113

[33] Wilson AD, Collins DR, Bingham GP (2005) Perceptual coupling in rhythmic movement coordination—Stable perception leads to stable action. Exp Brain Res 164:517–528 15887008 10.1007/s00221-005-2272-3

[34] Aickin M, Gensler H (1996) Adjusting for multiple testing when reporting research results: the Bonferroni vs Holm methods. Am J Public Health 86: 726–728 8629727 10.2105/ajph.86.5.726 PMC1380484

[35] Walker E, Nowacki AS (2011). Understanding equivalence and noninferiority testing. J Gen Intern Med 26(2):192–196 10.1007/s11606-010-1513-8 20857339 PMC3019319

